# CIP2A facilitates the G1/S cell cycle transition via B‐Myb in human papillomavirus 16 oncoprotein E6‐expressing cells

**DOI:** 10.1111/jcmm.13693

**Published:** 2018-06-12

**Authors:** Yonghao Tian, Hanxiang Chen, Lijun Qiao, Wenhao Zhang, Jingyi Zheng, Weiming Zhao, Jason. J. Chen, Weifang Zhang

**Affiliations:** ^1^ Department of Orthopedic Surgery Qilu Hospital of Shandong University Jinan Shandong China; ^2^ Department of Microbiology and Key Laboratory of Infection and Immunity of Shandong Province School of Basic Medical Sciences Shandong University Jinan Shandong China; ^3^ Cancer Research Center School of Basic Medical Sciences Shandong University Jinan Shandong China; ^4^ Department of Medicine University of Massachusetts Medical School Worcester MA USA

**Keywords:** B‐Myb, Cdk1, CIP2A, E6 oncoprotein, G1/S transition, human papillomavirus

## Abstract

Infection with high‐risk human papillomaviruses (HR‐HPVs, including HPV‐16, HPV‐18, HPV‐31) plays a central aetiologic role in the development of cervical carcinoma. The transforming properties of HR‐HPVs mainly reside in viral oncoproteins E6 and E7. E6 protein degrades the tumour suppressor p53 and abrogates cell cycle checkpoints. Cancerous inhibitor of protein phosphatase 2A (CIP2A) is an oncoprotein that is involved in the carcinogenesis of many human malignancies. Our previous data showed that CIP2A was overexpressed in cervical cancer. However, the regulation of CIP2A by HPV‐16E6 remains to be elucidated. In this study, we demonstrated that HPV‐16E6 significantly up‐regulated CIP2A mRNA and protein expression in a p53‐degradation‐dependent manner. Knockdown of CIP2A by siRNA inhibited viability and DNA synthesis and caused G1 cell cycle arrest of 16E6‐expressing cells. Knockdown of CIP2A resulted in a significant reduction in the expression of cyclin‐dependent kinase 1 (Cdk1) and Cdk2. Although CIP2A has been reported to stabilize c‐Myc by inhibiting PP2A‐mediated dephosphorylation of c‐Myc, we have presented evidence that the regulation of Cdk1 and Cdk2 by CIP2A is dependent on transcription factor B‐Myb rather than c‐Myc. Taken together, our study reveals the role of CIP2A in abrogating the G1 checkpoint in HPV‐16E6‐expressing cells and helps in understanding the molecular basis of HPV‐induced oncogenesis.

## 1 INTRODUCTION

Human papillomavirus (HPV) is a small DNA virus that replicates in the stratified layers of skin and mucosa and is one of the most common sexually transmitted infections. The high‐risk HPV type infections are associated with cervical carcinoma, which is one of the leading causes of cancer death in women worldwide.^1^ In addition, HPV infections are linked to more than 50% of other anogenital cancers and cancers of the oesophagus.^2^ Although tobacco and alcohol are responsible for most head and neck squamous cell carcinomas (HNSCCs), there is evidence for a causal association between HPV infections and HNSCCs. Despite a steady decrease in the number of overall HNSCCs cases in the past decades, the incidence of oropharyngeal cancer has increased significantly.^3^ Notably, in the meantime, the HPV DNA detection rate has increased from 16.3% to 71.7% in oropharyngeal cancer.^4^

Viral oncogenes have provided significant insights into important biological activities. HPV oncogenes E6 and E7 are consistently expressed in HPV‐positive cervical cancers,^5^ and the sustained expression of these genes is essential for the maintenance of the transformed state of HPV‐positive cells.^6^ E6 and E7 proteins promote the degradation of the tumour suppressors p53 and retinoblastoma protein (pRb), respectively, thus modulating multiple biological functions including immortalization of primary cells, transformation of mouse fibroblasts, tumorigenesis in animals, abrogation of cell cycle checkpoints and chromosomal instability.^7^, ^8^, ^9^ The ability of high‐risk HPV E6 protein to degrade p53 is thought to be a primary mechanism in inducing cellular transformation.

Cancerous inhibitor of PP2A (CIP2A) is an oncoprotein that was first identified as an endogenous physiological inhibitor of tumour suppressor protein phosphatase 2A (PP2A), a serine/threonine phosphatase.^10^ CIP2A is believed to execute its oncogenic functions mainly through stabilizing c‐Myc by inhibiting PP2A dephosphorylation of c‐Myc serine 62 (S62).^10^ Various independent studies have found that CIP2A is overexpressed in many types of human carcinomas, including breast, lung, gastric and hepatocellular cancers. In addition to the role of CIP2A in promoting cellular transformation and cancer aggressiveness, CIP2A is also associated with a high tumour grade (for a review see Ref.^11^). CIP2A is related to a poor patient prognosis and may be applied as a prognosis biomarker in evaluating the risk of tumour metastasis. In addition, it is overexpressed in 70% of most solid malignancies samples, while it is rarely expressed in normal tissues, which makes CIP2A a possible therapeutic target (for a review see Ref.^12^). Although the oncogenic role of CIP2A in human malignancies has been well elucidated, how it modulates cell proliferation and cell cycle remains largely unknown.

We have recently demonstrated that CIP2A is overexpressed and positively associated with HPV‐16E7 in cervical cancer tissues and cells, and the expression of CIP2A is correlated with tumour grade.^13^ However, as another important oncoprotein encoded by HPV, how 16E6 protein regulates CIP2A and the role of CIP2A in 16E6‐expressing cells remain unclear. In this report, we detected the mRNA and protein expression of CIP2A in 16E6‐expressing primary human keratinocytes and explored the role of CIP2A in cell proliferation and G1 checkpoint regulation. We showed that HPV‐16E6 protein up‐regulated CIP2A by degrading p53 in 16E6‐expressing cells and that CIP2A facilitated the G1/S transition by modulating Cdk1 and Cdk2 proteins in a B‐Myb–dependent manner.

## 2 MATERIALS AND METHODS

### 2.1 Cell culture and plasmids

Primary human keratinocytes (PHKs) were derived from neonatal human foreskins collected from the University of Massachusetts Hospital. PHKs were cultured on mitomycin‐treated J2‐3T3 mouse fibroblast feeder cells in F‐medium plus 5% foetal bovine serum (FBS) with all supplements as described previously.^14^ To alleviate the concern that PHKs have very low transfection efficiencies and transfection usually causes G1 arrest, we use human telomerase reverse transcriptase‐expressing retinal pigment epithelium (RPE1) cells expressing HPV‐16E6 for the siRNA experiments. RPE1 cells were originally obtained from Clontech^15^ and maintained in a 1:1 dilution of DMEM (Dulbecco's modified Eagle's medium) and Ham's F12 medium. The HPV‐16–positive cervical cancer cell line SiHa was purchased from the American Type Culture Collection (ATCC) and maintained in DMEM. All media were supplemented with 100 U/mL penicillin and 100 μg/mL streptomycin. All cells were grown in a humidified 5% CO_2_ atmosphere at 37°C.

Primary human keratinocytes and RPE1 cells stably expressing HPV‐16E6 or F2V (Phe‐2 to Val mutation), an E6 mutant defective in p53 degradation or empty pBabe‐puromycin vector, were established by retrovirus‐mediated infection. To ensure a high percentage of cells that expressed 16E6, F2V or contained the Babe vector, PHKs and RPE1‐derived cell lines were maintained with puromycin and used within 8 passages.

The pCDNA3 WT B‐Myb (#25965) plasmid was purchased from addgene.

### 2.2 RNA extraction, RT‐PCR and real‐time quantitative RT‐PCR (qRT‐PCR)

Total cellular RNA extraction was performed with the RNeasy mini kit (Qiagen). RNA was then reverse‐transcribed into cDNA with an iScript cDNA synthesis kit (Bio‐Rad). The cDNA samples were subjected to PCR amplification with the primers described previously^16^:

HPV‐16E6, For: 5′‐CTGCAATGTTTCAGGACCCA‐3′,
Rev: 5′‐CCTAATTAACAAATC‐3′.
β‐actin, For: 5′‐TGGCATTGCCGACAGGATGCAGAA‐3′,
Rev: 5′‐CTCGTCATACTCCTGCTTGCTGAT‐3′.

β‐Actin was used as a control for RNA loading and reverse transcription efficiency.

Quantitative RT‐PCR of CIP2A and cell cycle‐related proteins were performed as described previously.^13^ Glyceraldehyde‐3‐phosphate dehydrogenase (GAPDH) oligos were used as a control. Primer pairs used for qRT‐PCR were as follows:

CIP2A, For: 5′‐GAACAGATAAGAAAAGAGTTGAGCATT‐3′,
Rev: 5′‐CGACCTTCTAATTGTGCCTTTT‐3′;
Cdk1, For: 5′‐TTTTCAGAGCTTTGGGCACT‐3′,
Rev: 5′‐AGGCTTCCTGGTTTCCATTT‐3′;
Cdk2, For: 5′‐CAAGCTGCTGGATGTCATTC‐3′,
Rev: 5′‐AGCAGCTGGAACAGATAGCTCT‐3′;
Cdk4, For: 5′‐GCATCCCAATGTTGTCCG‐3′,
Rev: 5′‐AGGCAGCCCAATCAGGTC‐3′;
Cdk6, For: 5′‐TCTTGCTCCAGTCCAGCTAC‐3′,
Rev: 5′‐AGCAATCCTCCACAGCTCTG‐3′;
cyclin A2, For: 5′‐GAAGACGAGACGGGTTGCA‐3′,
Rev: 5′‐AGGAGGAACGGTGACATGCT‐3′;
cyclin B1, For: 5′‐AACTTTCGCCTGAGCCTATTTT‐3′,
Rev: 5′‐TTGGTCTGACTGCTTGCTCTT‐3′;
cyclin D1, For: 5′‐CCGTCCATGCGGAAGATC‐3′,
Rev: 5′‐ATGGCCAGCGGGAAGAC‐3′;
cyclin E1, For: 5′‐GGATGTTGACTGCCTTGA‐3′,
Rev: 5′‐CACCACTGATACCCTGAAA‐3′; and
GAPDH, For: 5′‐GCACCGTCAAGGCTGAGAAC‐3′.
Rev: 5′‐GCCTTCTCCATGGTGGTGAA‐3′.

The relative expression was defined by the comparative Ct value.

### 2.3 Western blot

Protein extracts were prepared in lysis buffer [10 mmol/L of Tris‐HCl (pH 7.4), 1% SDS, 1.0 mmol/L of sodium orthovanadate and protease inhibitors]. Fifty micrograms of protein from each cell lysate was denatured and separated by SDS‐PAGE and transferred onto a PVDF membrane. Membranes were probed with primary antibodies specific for the following proteins: Cdk1 (BD Biosciences, 610038), p21 (BD Biosciences, 610233); CIP2A (Novus Biologicals, NB100‐68264); B‐Myb (sc‐725), Cdk2 (sc‐6248), Cdk4 (sc‐260), Cdk6 (sc‐177), cyclin A2 (sc‐751), cyclin B1 (sc‐752), cyclin D1 (sc‐718), cyclin E1 (sc‐198), c‐Myc (sc‐764), p53 (sc‐126) and GAPDH (all from Santa Cruz Biotechnology, sc‐25778); and phospho‐c‐Myc (phospho S62, Abcam, ab51156), HPV‐16/18E6 (Abcam 70) and β‐tubulin (Sigma‐Aldrich, T4026). Horseradish peroxidase (HRP)‐conjugated antimouse or anti‐rabbit IgG (Santa Cruz Biotechnology) was used as secondary antibodies. An enhanced chemiluminescence kit (ECL, Pierce, USA) was used for detection.

ImageJ was used for the analysis of the grey values of the Western blot bands. We first opened the file and defined the individual sample as a rectangular box in which the band was fitted as tight as possible; then, we clicked the “analyze‐measure” and obtained the grey value. The same box was used to measure all of the bands in one file. The grey values of the corresponding loading control (GAPDH/β‐tubulin) were acquired following the same procedure. The grey value of the protein of interest was divided by the grey value of the corresponding loading control to get the relative value of each band. The relative value of the first band of the protein of interest in each image was set as “1,” and the relative values of the other bands were divided by the relative value of the first band to obtain a ratio, and the ratio was the “final relative value” of the band. When Western blot experiments were repeated 3 times, we obtained 3 ratios. These ratios were used for the calculation of mean and SD values.

### 2.4 Small interfering RNA and cell transfection

Chemically modified small interfering RNA (siRNA) oligonucleotides against CIP2A have been described previously,^13^ and the sequences of siRNA targeting CIP2A, B‐Myb or HPV‐16E6 (siRNA 209) are as follows:

CIP2A siRNA, 5′‐CUGUGGUUGUGUUUGCACUTT‐3′,
B‐Myb siRNA, 5′‐GAUCUGGAUGAGCUGCACU‐3′, described in Ref.^17^;
HPV‐16E6 siRNA 209, 5′‐UCCAUAUGCUGUAUGUGAU‐3′, described in Ref.^18^

A scrambled siRNA was synthesized for use as the control.

Retinal pigment epithelium or SiHa cells were seeded in 6‐cm dishes to ensure 30%‐50% confluence at the time of transfection and cultured in medium without antibiotics for 24 hours. siRNAs were transfected into cells at a final concentration of 20 nmol/L using Lipofectamine RNAiMAX (Invitrogen). For knockdown analysis, cells were harvested 48 hours after transfection, and protein levels were analysed by Western blot. CIP2A was knocked down approximately 70% after siRNA transfection, which was close to the level expressed in the Babe vector cells. For cell cycle analysis, 36 hours after transfection, cells were treated with bleomycin, incubated for an additional 24 hours and harvested.

### 2.5 Cell viability assay

The cell viability assay was assessed by use of the Cell Counting Kit 8 (CCK‐8, Dojindo, Japan) following the manufacturer's protocol.

### 2.6 FACS analysis

For cell cycle analysis, RPE1 or SiHa cells were treated with PBS or 10 μg/mL bleomycin for 24 hours. Cells were harvested, fixed in 70% ethanol at 4°C overnight and stained with 50 μg/mL propidium iodide (PI) supplemented with 70 μg/mL DNase‐free RNAse A for 30 minutes at room temperature and analysed by flow cytometry.

The 5‐bromo‐2′‐deoxyuridine (BrdU, BD Biosciences) labelling experiment was performed according to the manufacturer's recommendations. Immunofluorescent cells were analysed by use of FCS Express software. All flow cytometric experiments were repeated at least 3 times.

### 2.7 Luciferase assay

The human Cdk1 and Cdk2 promoter‐luciferase constructs—pGL3‐Cdk1 and pGL3‐Cdk2—were purchased from Biosune. The renilla luciferase control plasmid was purchased from Promega.

To evaluate whether B‐Myb regulates the transcription of Cdk1 and Cdk2, RPE1 cells were cotransfected with the Cdk1 or Cdk2 promoter‐luciferase constructs and renilla luciferase control plasmid together with the B‐Myb siRNA plasmid. Cells were harvested after 48 hours, and luciferase activity was measured with the dual‐luciferase reporter assay system following the manufacturer's recommendations.

### 2.8 Statistical analysis

All data are expressed as the means and standard deviations (SD). Statistical analyses were based on nonparametric tests and performed with SPSS for Windows software, version 12.0. In all analyses, *P* ≤ .05 was deemed significant.

## 3 RESULTS

### 3.1 High‐risk HPV E6 up‐regulates CIP2A in a p53‐degradation‐dependent manner

**Figure 1:**
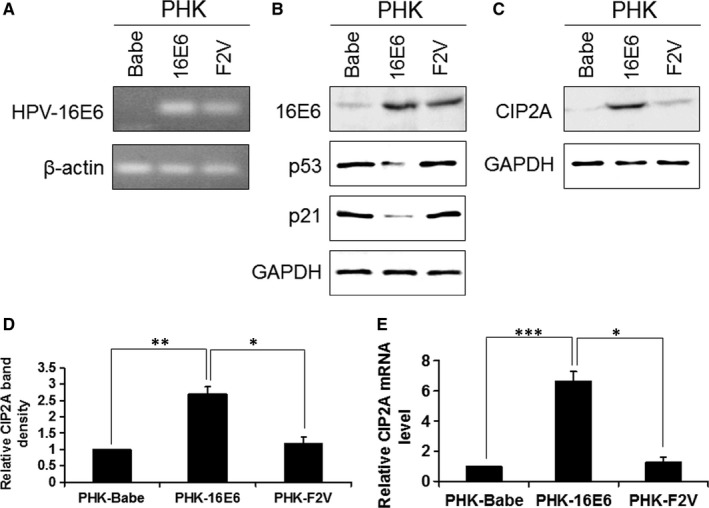
Induction of CIP2A mRNA and protein expression by HPV‐16E6 in PHKs. A, mRNA expression of HPV‐16E6 in PHKs expressing 16E6 and F2V using β‐actin as a loading control. B, Protein levels of HPV‐16E6, p53 and p21 in PHKs expressing 16E6 and F2V. Expression of GAPDH was used as a loading control. A representative of 2 independent experiments is shown. C, HPV‐16E6 expression leads to increased protein expression of CIP2A in PHKs. Data from a representative of 3 experiments are shown. D, Data from 3 experiments are summarized. E, Relative CIP2A mRNA expression was determined by qRT‐PCR in the above cells. Data from 3 experiments are summarized. The mean and standard deviation (SD) of 3 independent experiments are shown. Babe, pBabe‐puromycin vector. *, *P* < .05; **, *P* < .01; and ***, *P* < .001

To explore the mechanism by which E6 regulates CIP2A, we utilized retrovirus‐mediated successive infection to establish PHKs expressing wild‐type HPV‐16E6. The ability to promote the degradation of p53 protein has been suggested as a mechanism by which E6 protein from the high‐risk HPV types exhibits oncogenic activities, so a 16E6 mutant‐F2V (Phe‐2 to Val mutation) that does not degrade p53 yet retains 16E6 activities for immortalization of HMECs was constructed as we previously described.^19^ The expression of HPV‐16E6 and F2V mRNAs was confirmed by RT‐PCR with 16E6 primers (Figure 1A), while no expression of 16E6 mRNA was observed in the Babe vector cells. The expression of 16E6 and F2V proteins was confirmed using an HPV E6 antibody (Figure 1B). Both p53 and its target p21 protein expression levels were decreased in 16E6‐expressing PHKs, while in the 16E6 mutant F2V‐expressing cells, both p53 and p21 proteins remained nearly the same as in Babe vector cells (Figure 1B).

We then examined CIP2A protein and mRNA levels in PHKs expressing HPV‐16E6. We showed that the CIP2A protein level was significantly up‐regulated (approximately 2.7‐fold; *P* < .01) by HPV‐16E6 (Figure 1C,D). In addition, the CIP2A mRNA level was elevated approximately 6.7‐fold (*P* < .001) in PHKs expressing 16E6 (Figure 1E). However, there were no significant differences in CIP2A protein or mRNA expression between F2V‐expressing PHKs and Babe vector cells, which indicates that the up‐regulation of CIP2A by HPV‐16E6 is p53‐degradation–dependent.

### 3.2 Depletion of CIP2A inhibits cell viability and DNA replication in 16E6‐expressing cells

**Figure 2:**
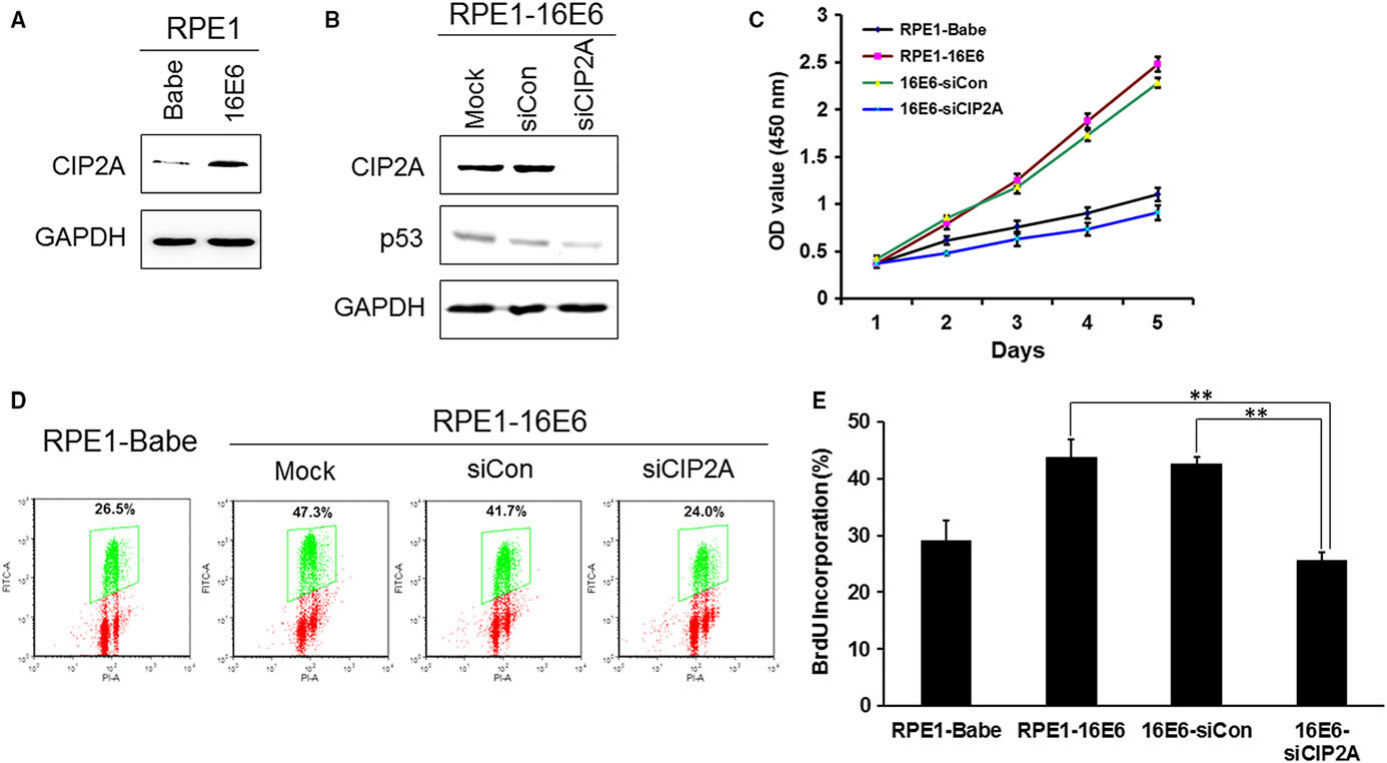
Inhibition of CIP2A by siRNA impeded cell viability and DNA synthesis in HPV‐16E6–expressing cells. A, Elevated expression of CIP2A protein in 16E6‐expressing RPE1 cells. B, Western blot analysis of CIP2A and p53 proteins after transfection with scrambled siRNA (siCon) or CIP2A siRNA (siCIP2A) for 48 h. A representative of 3 independent experiments is shown. C, Cell viability assay of RPE1‐16E6 cells with CIP2A knockdown. D, Representative flow cytometry of BrdU staining profiles is shown. E, The mean and SD of BrdU‐positive cells from 3 experiments are summarized. **, *P* < .01.

It has been reported that CIP2A promotes cell proliferation (for a review see Ref.^12^). To explore the role of CIP2A in cellular proliferation of 16E6‐expressing cells, small interfering RNA (siRNA) specific to CIP2A that was previously shown to effectively down‐regulate CIP2A expression was used.^13^ We have constructed HPV‐16E6–expressing RPE1 cells and confirmed the expression of 16E6 in these cells previously.^20^ Similar to what we have observed in PHKs, CIP2A protein expression was up‐regulated in RPE1 cells expressing 16E6 (Figure 2A). Depletion of CIP2A with siRNA efficiently decreased the CIP2A protein level in 16E6‐expressing cells. As HPV‐16E6 degrades p53, the steady‐state level of p53 is low in 16E6‐expressing cells. Knockdown of CIP2A further reduced the expression of p53 protein (Figure 2B). A CCK‐8 assay was performed to determine whether CIP2A had any effect on cell viability. As shown in Figure 2C, CIP2A knockdown inhibited the viability of 16E6‐expressing RPE1 cells.

To examine the effect of CIP2A on S phase entry and DNA replication in 16E6‐expressing cells, a 5‐bromo‐2′‐deoxyuridine (BrdU) incorporation approach was applied to mark cells in S phase. Compared with scrambled siRNA, knockdown of CIP2A by siRNA significantly decreased the number of BrdU‐positive cells (24.0% vs 41.7%) (*P* < .01) (Figure 2D,E). Therefore, CIP2A silencing hindered cells entering S phase and impaired DNA synthesis in HPV‐16E6–expressing cells.

### 3.3 CIP2A knockdown diminishes the ability of 16E6 protein to abrogate the G1 checkpoint

**Figure 3:**
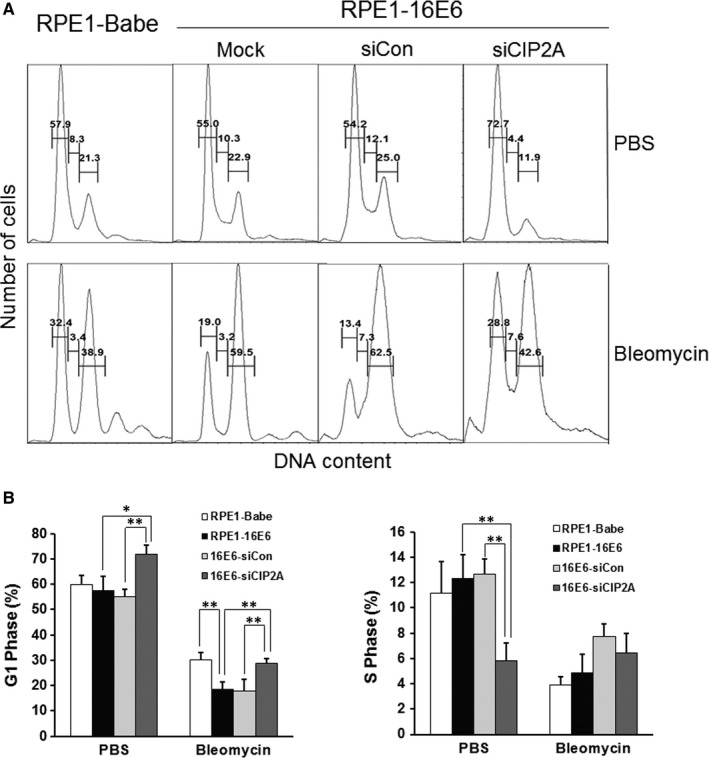
Silencing CIP2A caused G1 arrest in 16E6‐expressing cells. A, Flow cytometric analysis of 16E6‐expressing cells transfected with CIP2A siRNA for 36 h, treated with PBS or 10 μg/mL bleomycin for 24 h and then stained with PI. G1, S and G2 phases are indicated. Data from a representative of 4 experiments are shown. B, Quantification of percentages G1 phase and S phase cells. Data from 4 experiments are summarized. *, *P* < .05; **, *P* < .01

Flow cytometry was performed to investigate the effect of CIP2A on cell cycle distribution. It is well known that HPV‐16E6 abrogates the G1 cell cycle checkpoint (for a review see Ref.^8^). However, the role of CIP2A in regulating the G1 checkpoint in 16E6‐expressing cells has not previously been investigated. For this, we treated cells with bleomycin, a radiomimetic agent inducing DNA breaks and causing both G1 and G2 arrest.^21^ CIP2A siRNA increased the proportion of cells that arrested at the G1/S transition (71.7 ± 3.7% vs 55.2 ± 2.6%, *P* < .01), which showed more cells accumulated at the G1 peak (Figure 3A,B). Less accumulation of cells at S phase was also observed (5.8 ± 1.4% vs 12.7 ± 1.2%, *P* < .01). Upon induction of DNA damage by bleomycin, considerably fewer (18.5 ± 2.8% vs 29.9 ± 3.1%, *P* < .01) 16E6‐expressing cells were arrested in G1 phase compared to the Babe vector cells, which indicated that 16E6 bypassed bleomycin‐induced G1 arrest. Notably, inhibition of CIP2A by siRNA severely diminished the ability of 16E6 to abrogate the G1 checkpoint by causing more cells to arrest in G1 phase (28.6 ± 2.0% vs 17.7 ± 4.9%, *P* < .01). These results indicate a role for CIP2A in bypassing the DNA damage‐induced G1 cell cycle checkpoint in 16E6‐expressing cells.

### 3.4 CIP2A knockdown down‐regulates Cdk1 and Cdk2 proteins

**Figure 4:**
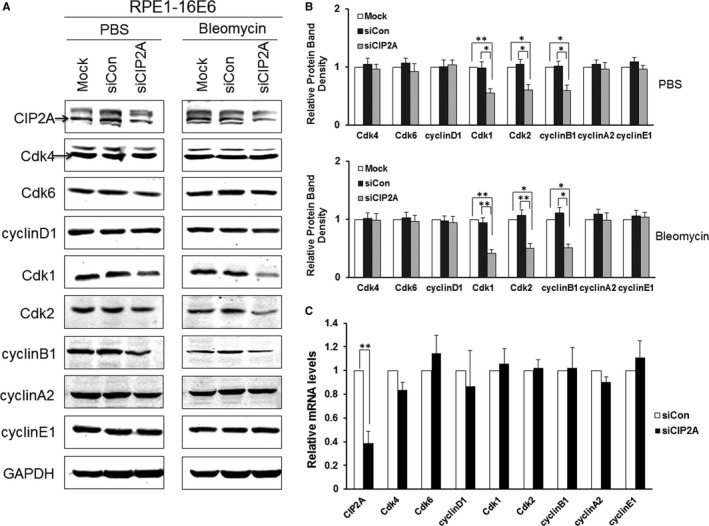
Silencing CIP2A caused decreased Cdk1 and Cdk2 proteins in 16E6‐expressing cells. A, Western blot analysis of CIP2A, Cdk4, Cdk6, cyclin D1, Cdk1, Cdk2, cyclin B1, cyclin A2 and cyclin E1 protein levels in cells expressing HPV‐16E6 transfected with CIP2A siRNA and then treated with PBS or 10 μg/mL bleomycin for 24 h. A representative of 3 independent experiments is shown. B, Quantification of all cell cycle‐related proteins. Data from 3 experiments are summarized. C, Relative mRNA levels of all cell cycle‐related genes determined by qRT‐PCR. Data from 3 experiments are summarized. *, *P* < .05; **, *P* < .01

Cell cycle progression is monitored by several checkpoints, among which the G1 checkpoint is very important, as it determines whether cells enter S phase and initiate DNA replication. To explore how CIP2A modulates the G1 checkpoint, we examined the expression of cell cycle‐related proteins including Cdk4, Cdk6, Cdk1, and Cdk2 and their associated cyclin partners—cyclin D1, cyclin A2, cyclin B1 and cyclin E1. In addition to Cdk2, which was proposed to be a major regulator in S phase entry, the protein level of the mitotic Cdk‐Cdk1 was also markedly reduced with CIP2A knockdown (*P* < .05) (Figure 4A,B). The cyclin partner of Cdk1‐cyclin B1 decreased significantly as well (*P* < .05). In contrast, no significant alterations were observed in the expression of structurally and functionally similar Cdk4 and Cdk6 proteins as well as their main partner cyclin D1 after CIP2A silencing. Meanwhile, no decrease in cyclin E1 and cyclin A2 was observed in CIP2A knockdown cells (Figure 4A,B). We then treated the cells with bleomycin and detected the expression of cell cycle‐related proteins, and similar alterations, such as Cdk1, Cdk2 and cyclin B1 expression decreasing significantly after CIP2A knockdown, were observed (Figure 4A,B). As Cdk2 and Cdk1 are the key factors to drive the G/S transition, these results hint that CIP2A might bypass the G1 checkpoint by regulating Cdk1 and Cdk2 proteins in 16E6‐expressing cells.

We performed qRT‐PCR to detect the mRNA levels of all cell cycle‐related genes. Interestingly, knockdown of CIP2A did not cause significant changes in mRNA levels of these cell cycle regulators (Figure 4C), which suggests that CIP2A does not affect the transcription of these genes and that the regulation of Cdk1 and Cdk2 by CIP2A may be post‐transcriptional.

### 3.5 Regulation of Cdk1 and Cdk2 by CIP2A depends on B‐Myb, instead of c‐Myc

**Figure 5:**
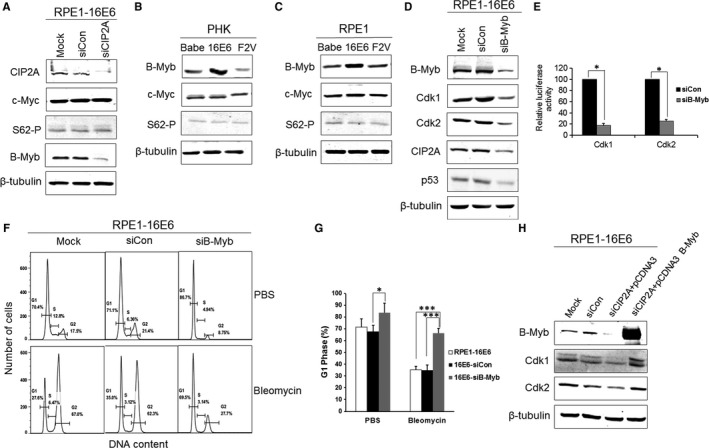
Regulation of Cdk1 and Cdk2 by CIP2A is dependent on B‐Myb rather than c‐Myc. A, Western blot analysis of CIP2A, c‐Myc, phospho‐S62‐Myc and B‐Myb protein levels in 16E6‐expressing cells after CIP2A knockdown. β‐Tubulin was used as a loading control. B, Protein levels of B‐Myb, c‐Myc and phospho‐S62‐Myc in 16E6‐expressing PHKs and (C) RPE1 cells. D, Protein levels of B‐Myb, Cdk1 and Cdk2, CIP2A and p53 in 16E6‐expressing cells after B‐Myb knockdown with siRNA. Data from a representative of 3 experiments are shown. E, Knockdown of B‐Myb down‐regulates Cdk1 and Cdk2 luciferase reporter activities. RPE1 cells were cotransfected with the Cdk1 or Cdk2 promoter‐luciferase constructs and renilla luciferase control plasmid together with B‐Myb siRNA plasmid. Cells were harvested after 48 h, and lysates were assayed for luciferase activity. F, Flow cytometric analysis of 16E6‐expressing cells transfected with B‐Myb siRNA treated with PBS or bleomycin. G1, S and G2 phases are indicated. A representative flow cytometry of 3 independent experiments is shown. G, Quantification of percentages G1 phase cells. Data from 3 experiments are summarized. H, Western blot analysis of B‐Myb, Cdk1 and Cdk2 in B‐Myb–overexpressing CIP2A knockdown cells. Data from a representative of 3 experiments are shown. *, *P* < .05; ***, *P* < .001

The transcription factor c‐Myc is a basic helix‐loop‐helix leucine zipper protein that is involved in cell proliferation, cell cycle progression, metabolism and malignant transformation through targeting of downstream genes.^22^ We speculated that cell cycle regulation by CIP2A may depend on c‐Myc as CIP2A stabilizes c‐Myc by preventing dephosphorylation on S62 of c‐Myc. Surprisingly, both the proteins of c‐Myc and phospho‐S62‐Myc remained unaltered following CIP2A silencing in HPV‐16E6–expressing cells (Figure 5A). Interestingly, CIP2A knockdown caused an obvious decrease in the steady‐state level of transcription factor B‐Myb (Figure 5A). We further examined the expression of B‐Myb and c‐Myc in PHKs expressing HPV‐16E6. As shown in Figure 5B, HPV‐16E6 greatly up‐regulated the expression of B‐Myb, while neither the expression of c‐Myc nor phospho‐S62‐Myc increased in 16E6‐expressing cells. Similar results were observed in 16E6‐expressing RPE1 cells (Figure 5C). Thus, the expression of CIP2A and B‐Myb but not c‐Myc was positively correlated in 16E6‐expressing cells. B‐Myb was reported to transactivate the transcription of Cdk1.^23^ To clarify the regulation of Cdk1 and Cdk2 by B‐Myb in 16E6‐expressing cells, we further knocked down B‐Myb with a specific siRNA, and correspondingly, markedly decreased expression of Cdk1 and Cdk2 was observed (Figure 5D). In addition, knockdown of B‐Myb caused reductions in CIP2A and p53 proteins, which suggests that there may be positive feedback between CIP2A and B‐Myb. We further determined the effect of B‐Myb on the transcription of Cdk1 and Cdk2‐luciferase constructs. Figure 5E shows that the activities of the Cdk1 and Cdk2 promoters were clearly reduced when B‐Myb was knocked down (*P* < .05). Moreover, knockdown of B‐Myb caused G1 arrest (83.8 ± 8.1% vs 67.8 ± 5.1%, *P* < .05), and there were fewer cells in S phase (2.20% vs 6.36%). When treated with bleomycin, B‐Myb knockdown caused G1 arrest (66.5 ± 3.8% vs 34.8 ± 4.7%, *P* < .001) (Figure 5F,G). Furthermore, we introduced a plasmid encoding B‐Myb in CIP2A knockdown cells, and the overexpression of B‐Myb rescued the expression of Cdk1 and Cdk2 (Figure 5H). Taken together, these experiments suggest that the regulation of Cdk1 and Cdk2 by CIP2A may rely on the transcription factor B‐Myb.

**Figure 6:**
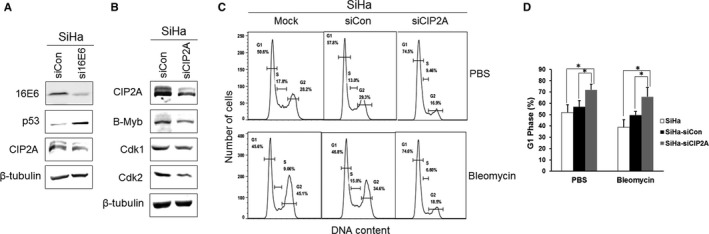
Inhibition of Cdk1 and Cdk2 by CIP2A knockdown in cervical cancer SiHa cells caused G1 arrest. A, Western blot analysis of 16E6, p53 and CIP2A after HPV‐16E6 knockdown in cervical cancer SiHa cells. B, Protein expression of CIP2A, B‐Myb, Cdk1 and Cdk2 in SiHa cells after CIP2A knockdown. Data from a representative of 3 experiments are shown. C, Flow cytometric analysis of SiHa cells with CIP2A knockdown treated with PBS or bleomycin. A representative flow cytometry of 3 independent experiments is shown. D, Quantification of percentages G1 phase cells. Data from 3 experiments are summarized. *, *P* < .05

To further verify the regulation and function of CIP2A in cervical cancer cells, we introduced HPV‐16–positive SiHa cells. HPV‐16E6–specific siRNA was reported to be effective and specific when transfected into SiHa cells.^18^ The decreased 16E6 protein and elevated p53 protein indicated that HPV‐16E6 was efficiently knocked down (Figure 6A). Our data showed that CIP2A was reduced after 16E6 knockdown. However, we noticed that the decrease in CIP2A was not dramatic, and we propose that this is mainly because HPV E7 also has the ability to up‐regulate CIP2A.^13^ Furthermore, we showed that B‐Myb might be a downstream target of CIP2A as CIP2A silencing greatly inhibited the expression of B‐Myb in SiHa cells (Figure 6B). As expected, the levels of Cdk1 and Cdk2 proteins decreased correspondingly. Flow cytometric analysis showed that CIP2A silencing caused more cells to arrest in G1 phase (71.9 ± 5.0% vs 56.9 ± 5.6%, *P* < .05) and fewer cells to enter S phase (9.46% vs 13.0%) (Figure 6C,D). Moreover, CIP2A silencing enhanced bleomycin‐induced G1 arrest, which showed a more accumulated G1 phase (65.8 ± 8.1% vs 49.7 ± 3.1%, *P* < .05) in SiHa cells. The data further hint that CIP2A may be involved in the G1 checkpoint by regulating Cdk1 and Cdk2 in a B‐Myb–dependent manner in cervical cancer cells.

## 4 DISCUSSION

By retrovirus‐mediated overexpressing of HPV‐16E6 and E6 mutant‐F2V, on the one hand, we showed that 16E6 significantly increased CIP2A mRNA and protein expression in a p53‐degradation–dependent manner. On the other hand, knockdown of CIP2A caused a reduction in the expression of p53 in HPV‐16E6‐expressing cells. Although the interaction between p53 and CIP2A needs to be further investigated, our finding is in accordance with that of previous studies. One study demonstrated that in human breast cancer tissues, CIP2A mRNA expression was positively correlated with p53 mutations; in addition, in breast cancer mouse models with p53 depletion, CIP2A protein expression was induced.^24^ Another study showed that after treatment with doxorubicin, CIP2A expression was increased in MDA‐MB231 (mutant p53 breast cancer cell line) cells and decreased in MCF‐7 (wild‐type p53 breast cancer cell line) cells, which demonstrated that cells with mutant p53 have higher CIP2A expression.^25^ Notably, treatment with p53 activator molecules resulted in a reduction in CIP2A expression in breast cancer cells.^26^ Taken together, our data and results from these investigations strongly suggest a negative association between p53 and CIP2A proteins, and further research will focus on the underlying molecular basis.

Genomic instability is an important prerequisite and hallmark of carcinogenesis. Cell cycle progression is monitored at several cell cycle checkpoints by regulating the expression and activity of cyclins and Cdks, whose defects contribute to genomic instability.^27^ The G1 checkpoint is the first to determine whether cells enter the S phase and initiate DNA replication. The G1 checkpoint is mainly regulated by Cdk2/cyclin E‐cyclin A complexes by phosphorylation of pRb.^28^ Upon DNA damage caused by exposure to genotoxic agents such as UV or chemicals, p53 is activated via multiple mechanisms and inactivates the Cdk2/cyclin E‐cyclin A complexes by activating transcription of the Cdk inhibitor p21, resulting in pRb hypophosphorylation and G1 cell cycle arrest.^29^ Because of the central role p53 plays in G1 checkpoint control and the efficient degradation of p53 by high‐risk HPV E6 protein, it is reasonable that HPV‐16E6 abrogates the G1 cell cycle checkpoint.^30^ However, whether CIP2A is involved in the abrogation of the G1 checkpoint in HPV‐16E6–expressing cells is unclear. Our data showed that knockdown of CIP2A by siRNA abrogated the ability of 16E6 to bypass the G1 checkpoint, thus proving the function of CIP2A in G1 checkpoint regulation.

Cdk2 was believed to be a major regulator and was essential in S phase entry. However, an increasing number of studies have shown that Cdk1 plays a vital role in the maintenance of cell cycle progression. A previous report showed that Cdk1 can substitute for Cdk2 during the G1/S transition.^31^ Moreover, a study of Cdk2/Cdk3/Cdk4/Cdk6‐knockout mice demonstrated that Cdk1 binds all cyclins and drives the whole cell cycle progression.^32^ Our data showed that both Cdk2 and Cdk1 function in the G1/S transition and reduced Cdk2 and Cdk1 by CIP2A knockdown lead to G1 arrest. Our results reveal that CIP2A facilitates the G1/S transition by regulating Cdk1 and Cdk2.

c‐Myc is a transcriptional factor implicated in control of cell proliferation as well as a variety of cellular regulatory processes. The regulation of c‐Myc by HPV‐16E6 is conflicting. HPV‐16E6 has been reported to activate and up‐regulate the expression of c‐Myc protein in a p53‐dependent manner.^33^ However, one study showed that HPV‐16E6 did not up‐regulate c‐Myc, and c‐Myc was not required for the activation of telomerase.^34^ Moreover, other report showed in vitro degradation of c‐Myc protein by 16E6.^35^ Therefore, the activation or degradation of c‐Myc by 16E6 may be cell‐specific or merely reflect the rate of cell proliferation under distinct environmental conditions. Our studies could not find a significant up‐regulation of c‐Myc in 16E6‐expressing PHKs, which is consistent with the results found in HFK (human foreskin keratinocytes, also named PHK).^34^ More importantly, CIP2A silencing did not repress either c‐Myc or phospho‐S62‐Myc expression, which indicates that the regulation of the G1 checkpoint by CIP2A is c‐Myc–independent.

B‐Myb is a transcription factor with specific DNA‐binding activity.^36^ Previous studies have shown that B‐Myb is overexpressed in cervical cancers.^37^ Importantly, the expression of B‐Myb mRNA peaks during the G1/S phase transition.^38^ In addition, overexpression of B‐Myb bypassed p53‐induced G1 arrest.^39^ Notably, B‐Myb was reported to bind to the Cdk1 promoter and activate the expression of Cdk1.^23^ It is well documented that B‐Myb was up‐regulated in HPV E7‐expressing cells.^40^ However, the expression and function of B‐Myb in 16E6‐expressing cells have not been reported. Our data demonstrated that B‐Myb protein was up‐regulated by HPV‐16E6, while CIP2A knockdown caused inhibition of B‐Myb. Although B‐Myb is involved in p53‐induced G1 arrest abrogation,^39^, ^41^ the underlying mechanism is not fully understood. We showed that B‐Myb was required for CIP2A to regulate Cdk1 and Cdk2. Our results suggest that positive regulation of Cdk1 and Cdk2 by B‐Myb contributes to bypassing the G1 checkpoint in 16E6‐expressing cells. Future research will focus on the precise regulation of B‐Myb by CIP2A.

We demonstrated that CIP2A is up‐regulated by HPV‐16E6 in a p53‐dependent manner. CIP2A is essential to abrogate the G1 cell cycle checkpoint by modulating Cdk1 and Cdk2 expression in HPV‐16E6–expressing cells. Furthermore, a luciferase assay showed that the regulation of Cdks by CIP2A in 16E6‐expressing cells depends on the transcription factor B‐Myb rather than c‐Myc. Our results reveal a specific role of CIP2A in HPV‐16E6–mediated regulation of G1/S cell cycle progression. Previous study demonstrated that CIP2A protein was specifically expressed in cervical cancer tissues and was undetectable in normal adjacent cervical tissues.^42^ Our laboratory found that CIP2A protein was overexpressed in cervical cancer and high grade of cervical intraepithelial neoplasia (CIN), CIN III, which was the key step from the precancerous to invasive cancer, but not in normal cervical, CINI or CINII tissues.^13^ This expression pattern indicates that CIP2A has a clinical prominence in the development of cervical cancer and makes CIP2A to be a potential diagnostic biomarker. In addition, CIP2A mutant mouse models showed no obvious abnormality in mouse development and viability.^43^ Therefore, the expression specificity and limited toxicity indicate that CIP2A inhibitors may be a hopeful therapeutic strategy for treatment of cervical cancer. However, much effort is needed for translational medicine and clinical application of targeting CIP2A for cervical cancer diagnosis and treatment.

## CONFLICT OF INTEREST

The authors confirm that there are no conflict of interests.
